# California

**Published:** 1875-07

**Authors:** 

**Affiliations:** San Jose, Cal.


### CALIFORNIA.

A LETTER FROM DR. GLEASON.

We have received the following graphic and characteristic letter from our friend, Dr. S. O. Gleason, and take the liberty of publishing it. The Doctor may not thank us for pressing him into the service of the BISTOURY, but then our readers will:

SAN JOSE, CAL., MAY 7, '75.

MY DEAR DOCTOR:

This is a gay old country. There is no place like it on the face of all this earth. These beautiful days—the fine refreshing sleep, in the cool nights, helps to renew old chaps like me, and make me feel young again.

This is the best country for *real out-of-door life* I have ever seen. From the first of May till the first of November you can take your blankets, gun and fishing-rod, ride all over the state, sleep where night overtakes you, with no fear of wet or cold. Can shoot all the game you want out of your carriage, or by going on foot a very little distance, can stop by some mountain stream and catch all the fine trout you wish. You get for game, common quail; mountain quail—much larger—very fine bird; mountain squirrels—a very fine gray; cotton tail rabbits; jack rabbits—with ears as long as a mule's, legs same proportion—jump! (oh, how they *will* jump!); the road runner—run like a streak—a curious bird. The jay, here, and the flicker, differ in color from our birds. The meadow lark is larger; ducks are small—cinnamon color. In the season you can shoot all the geese your wagon will hold, in one day. Went up into the mountains north of Santa Barbara, to a "bee ranch," and staid several days in camp.—Had some genuine sport—could find game at any time and in any quantity that would satisfy any reasonable desire on the part of any sportsman. Saw one day, within easy range for your rifle, two fine bucks, but as I was out hunting quail, was not prepared for such game. An old grizzly came within 100 yards of camp and ate up two swarms of bees, or rather their honey. His tracks measured 13 to 14 inches in the sand.

One of the men in camp, shot a California lion only a few days before I went there.

You would enjoy this fine climate, fine sporting, fine camping, fine scenery, fine sky and fine air. You can keep warm and dry all the time. When you get time for a *real good time*, this is the country for fun and enjoyment.

There are more rich men in California, in proportion to inhabitants, than any other country. Men get rich here, as it were, in a day. They often get poor also. There exists much insanity here. The excitements of this country are great —the climate is highly stimulating—the operators in stocks, real estate, or mining, are *bold* and often *hazardous* in the extreme. The best real estate is in the hands of a few men ; much has been purchased from the spaniards for small sums—they will only sell at high figures, hence it is not a good place for emigrants with small capital ; too many are coming here this spring. There are numerous very bold stage robberies—they occur almost every week even on traveled routes. The country is so wild, mountain passes so frequent, that it affords fine chances for real banditti to flourish; murder and suicide are frequent. They go in here for "big things," right or wrong. It is a fast country.

It is a great country of extremes. You find edens and deserts—you find scanty timber, and the most royal forests in all the world.

You find the rich and the poor, the real poor. All races of men—all the habits, customs and national peculiarities of all races, even to the genuine "heathern chinee." You can hear the German, Spanish, French and Chinese languages, all in five minutes walk. Society is as yet not consolidated, —staid—fixed ; but is improving very fast. In the streets you will see a fine modern house, and then, an old spanish adobe of one story—long and narrow, with its old tile roof. The Spanish senorita will come out of one front gate and an American belle out of the next ; the former with cigarette, the latter with her smelling bottle. But the most villainous looking humans are a mixture of Mexican and digger indian; they look *born* cut-throats, every inch of them.

There are larger and more beautiful flowers here than I have ever seen in any place. Roses ! oh, roses ! they make one almost wild with delight. Such fucias, such geraniums, such floral splendors fairly daze one, and fill him full of delight, beyond compare.

The peach, apple, cherry, pear, apricot, olive, almond, prune, english walnut, orange and lemon, all grow here finely. Strawberries ! oh, such berries ! such vast fields of them, too, attended by chinese, are a feature in this valley. They are only fifteen cents per pound at this time, and will soon be bought for ten cents. We have all we want every day.

This is a fine place to enjoy life in, if one has the "needful" before he comes. There can be more real comfort gotten out of life here, because the climate permits one to be so much out of door. You would enjoy it here, hugely. The weather is so fine *every day*, that nobody notices it at all. You say "it is a fine day," it almost provokes a smile—as much as to say, "we don't have any other kind."

Well, under this sheet is a copy of the BISTOURY. Have read it—was as welcome as the face of an old friend.

We leave here the 10th inst., for the Calavarus big trees and for the Yosemite valley. We expect to have a tedious, but at the same time, an interesting trip. Wish you and Mrs. U. could go in with us—we could enjoy it hugely.

This state abounds in the grand and sublime—the beautiful and romantic. You cannot go in any direction and not be charmed with the scenery. Every place pleases you in many of its features.

Mark Twain did not exaggerate the language of California, in "Roughing It."

When you are invited to take a drink, they say, "Nominate your poison—what will you have—tarrantula juice, seven days sickness, or instant death?" So they go it, on extremes of expression. They ride fast, drive fast, go it fast. They go in for fun, frolic and enjoyment.

Well, "adios, senor," as the spaniards say. This is a queer mixture for a letter—but it is a queer country of which I write.

Hope you are *warm* by this time.

Truly your friend, S. O. G.

---

MEXICAN BLEEDING.—Mr. H. H. Bancroft, in the recently published first volume of his work on the aboriginal races of Western America, mentions a quaint custom of a Mexican tribe, which is to medical science what the method of roasting pigs practiced by Lamb's mythical Chinese was to cooking. When these ingenious persons wish to bleed a patient, the doctor shoots at him with a bow and arrow, the arrow being guarded at a certain distance from the point, until he has the good luck to strike a satisfactory vein. If our doctors have not learnt how to cure diseases, we must admit that they are able to inflict wounds with a smaller superfluity of injury.

